# Factors influencing the caregiver burden for family caregivers of people with moderate-to-severe dementia: A structural equation modeling analysis

**DOI:** 10.1371/journal.pone.0341719

**Published:** 2026-02-06

**Authors:** Hong Qi, Jingjing Ban, Nana Wang, Tian Yao, Li Li, Shan Tang

**Affiliations:** 1 School of Nursing, Shanxi Medical University, Taiyuan, China; 2 Department of Gastrointestinal Surgery, First Hospital of Shanxi Medical University, Taiyuan, China; 3 Department of Neurology, First Hospital of Shanxi Medical University, Taiyuan, China; 4 Nursing Department, First Hospital of Shanxi Medical University, Taiyuan, China; University of Sao Paulo, BRAZIL

## Abstract

**Background:**

Dementia poses an increasingly serious public health challenge worldwide, particularly in China, where home-based care remains the primary form of management. Family caregivers of older adults with moderate-to-severe dementia often experience a substantial caregiving burden, which can adversely affect both their own well-being and that of the patients.

**Objective:**

This study aimed to examine the level of caregiver burden among family caregivers of older adults with moderate-to-severe dementia and to explore the relationships between patient activities of daily living (ADL), quality of life (QoL), depression severity, caregiver QoL, depression severity, and caregiver burden.

**Methods:**

A cross-sectional study in 22 tertiary general hospitals in Shanxi Province, China, involved 529 dyads of older adults with moderate-to-severe dementia and their family caregivers. Caregivers completed the Zarit Burden Interview, the Patient Health Questionnaire-9, and the World Health Organization Quality of Life–Brief Version. Older adults completed the Patient Health Questionnaire-9, the Activity of Daily Living Scale, and the Quality of Life in Alzheimer’s Disease scale. Structural equation modeling evaluated relationships among patient ADL, QoL, depression severity, caregiver QoL, depression severity, and caregiver burden.

**Results:**

Patient QoL, ADL, and caregiver QoL had direct negative effects on caregiver burden, whereas patient and caregiver depression severity had direct positive effects on caregiver burden. Meanwhile, caregiver QoL partially mediated the relationships between patient QoL, depression severity, ADL, caregiver depression severity, and caregiver burden.

**Conclusion:**

The results indicate that higher patient QoL, better patient ability in ADL, higher caregiver QoL, and lower levels of depressive severity in both patients and caregivers are associated with reduced caregiver burden. Healthcare professionals should implement family-centered, comprehensive interventions to alleviate caregiver burden.

## Introduction

With the rapid aging of the global population, dementia has become a major public health issue and poses a substantial challenge to global public health. According to a recent report by the World Health Organization, an estimated 55 million people worldwide are living with dementia, and a new case is diagnosed every three seconds [1]. This number is expected to rise to 152 million by 2050 [2]. China has a particularly high prevalence of dementia, accounting for roughly one quarter of all cases globally [3]. Globally, up to 84% of people with dementia receive care at home [4]. In China, because of the relatively underdeveloped social health care and eldercare service systems, as well as the influence of traditional cultural values, about 80% of individuals with dementia are cared for at home by their family members [5].

Dementia is a progressive neurodegenerative condition that leads to worsening impairments in memory, language, behavior, and the performance of everyday activities [6]. Among older adults with dementia, family caregivers—typically spouses or adult children—provide ongoing, unpaid support. They play a pivotal, yet often underrecognized, role in meeting the daily needs of these individuals, taking responsibility for tasks such as grocery shopping, organizing and supervising medication use, managing financial and legal affairs, preventing wandering, and assisting with both basic and instrumental activities of daily living (ADL) [7]. As dementia progresses, affected individuals become increasingly dependent on their caregivers [8]. This dependence is especially pronounced among those with moderate-to-severe dementia, whose capacity to carry out everyday activities progressively declines, thereby imposing substantial psychological, emotional, physical, and financial burdens on caregivers [9,10].

Caregiver burden is defined as the extent to which caregivers perceive that their emotional and physical health, social life, and financial situation are negatively affected by caring for a relative [11]. Existing studies have shown that caregiver burden is influenced by multiple factors, which can be broadly categorized into patient-related, caregiver-related, and social factors. Patient-related factors mainly include disease severity [12], self-care ability [13], Behavioral and Psychological symptoms [14], cognitive function [15], memory and behavioral problems [16], and quality of life (QoL) [17], among others. Caregiver-related factors primarily include health literacy [18], psychological status [15,18], general health status [19], and level of empathy [20]. Social factors are mainly reflected in the level of social support [15,21].

Previous studies on the factors influencing caregiver burden among family caregivers of older adults with dementia have largely focused on relatively narrow, single dimensions. Comprehensive investigations that simultaneously integrate patient-related, caregiver-related, and social factors, as well as systematic analyses of the underlying mechanisms, remain limited. Given that caregiver burden is shaped by the joint influence of multiple dimensions, developing an integrative theoretical model that incorporates these factors and examines their potential pathways may help identify key points of influence and potential targets for intervention, thereby providing a basis for strategies to reduce caregiver burden. Therefore, this study employed structural equation modeling to propose a hypothetical model of caregiver burden among family caregivers of older adults with moderate-to-severe dementia and to test the path relationships among relevant factors, to deepen understanding of the mechanisms underlying caregiver burden.

The stress process model, proposed by Pearlin in 1990, posits that various factors influence both exposure to stress and individuals’ coping responses. Some of these factors function as natural protective personal resources that can buffer or mitigate the negative effects of stress, whereas other related factors may amplify the impact of stress, making individuals more vulnerable to its adverse consequences. The model suggests that caregiving outcomes are shaped by subjective and objective stressors, role strains, and psychological strains, and are balanced through mediating factors such as coping strategies and social support resources derived from family, friends, and other social networks [22].

The World Health Organization broadly defines QoL as an individual’s perception of their position in life within the context of the culture and value systems in which they live, and in relation to their goals, expectations, standards, and concerns [23]. As dementia progresses, patients experience a gradual decline in QoL and increasingly require specialized care in their daily activities [24]. During disease progression, even brief lapses in supervision may result in adverse events, such as falls, getting lost, or unintentionally harming others. However, the intensive nature of caregiving tasks, combined with patients’ low level of cooperation, makes the provision of care particularly challenging. As a result, caregivers experience increasing levels of stress, which can have detrimental effects on their physical and mental health [25].

ADL are commonly divided into basic ADL, which involve self-care skills such as bathing, dressing, and eating, and instrumental ADL, which encompass more complex tasks such as medication management, grocery shopping, and handling finances [26]. A longitudinal study by Kawaharada et al. showed that the degree of decline in ADL among patients with dementia was significantly and positively associated with caregiver burden; that is, the more severe the functional decline, the greater the stress and burden experienced by caregivers [27].

In addition to cognitive decline, approximately 90% of patients with dementia experience behavioral and psychological symptoms, including psychosis, aggression, agitation, and depression [28]. Notably, compared with age-matched controls, patients with dementia are about twice as likely to be diagnosed with major depressive disorder [29]. Approximately 16% of individuals with dementia receive a concurrent diagnosis of major depressive disorder, whereas about 32% exhibit depressive symptoms without meeting the full diagnostic criteria for major depressive disorder, as part of the neuropsychiatric symptom cluster of dementia [30–32]. In people with dementia, depressive symptoms may present clinically as somatic manifestations (e.g., loss of appetite, low energy) and behavioral changes (e.g., irritability, social withdrawal, sadness) [33]. Although individuals with dementia and depressive symptoms may not fulfill all the criteria for major depressive disorder, these symptoms can still substantially affect both patients and their caregivers [34]. In individuals with dementia, depressive symptoms are linked to a range of unfavorable outcomes, including poorer QoL, declines in functional ability, and elevated mortality risk, as well as increased psychological strains and burden among caregivers [34–38].

Meanwhile, caregivers of people with dementia exhibit a higher rate of depression compared with caregivers of individuals with other illnesses or disabilities [39]. Moreover, this chronic stress may trigger a variety of psychological and physical problems and increase the risk of suicide among caregivers [40]. It has been shown to impair caregivers’ physical health, reduce their QoL, and hasten the institutionalization of people with dementia into long-term care facilities [41].

Social support refers to the adequacy and quality of assistance that individuals receive from their social networks—such as family members, friends, and other significant persons—when they need help. As a positive external resource, social support may enable family caregivers to provide care to individuals with disabilities more effectively, thereby reducing caregiver burden and lowering the risk of adverse health outcomes [21]. Previous studies have shown that higher levels of perceived social support are associated with better QoL among family caregivers [42,43]. Therefore, in this study, caregiver QoL was used as a comprehensive outcome indicator to capture the overall positive impact that perceived social support may exert on caregivers’ physical and mental health, functional status, and overall well-being.

**Fig 1:**
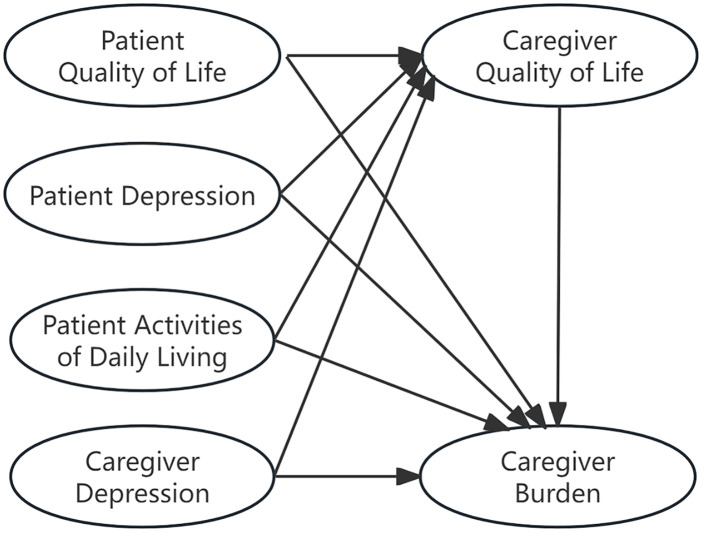
Hypothetical model.

Based on the stress process model, we constructed a hypothetical model for this study (Fig 1) and proposed the following hypotheses: H1: Family caregivers of older adults with moderate-to-severe dementia in Shanxi Province, China, experience a measurable level of caregiver burden. H2: Higher patient QoL, better patient ADL, lower patient depression severity, higher caregiver QoL, and lower caregiver depression severity are associated with lower caregiver burden. H3: The relationships between patient QoL, ADL, depression severity, caregiver depression severity, and caregiver burden are mediated by caregiver QoL.

## Materials and methods

### Participants and recruitment

The study was conducted from March 25 to September 25, 2025, and employed a hospital-based stratified sampling strategy to recruit older adults with moderate-to-severe dementia and their family caregivers. Shanxi Province comprises 11 prefecture-level cities. Based on feasibility, case volume, and departmental capacity, two tertiary general hospitals were selected from each city as study sites, yielding a total of 22 participating hospitals. Using neurology department records from January 1 to December 31, 2024, we calculated the proportion of patients with moderate-to-severe dementia at each center relative to the total number of such patients across all centers during the same period and allocated the target sample size to each hospital proportionally. During the study period, each center prospectively and consecutively screened eligible patients and compiled a registry. Patients were assigned serial numbers according to the order in which they were identified. Within each hospital, systematic sampling was then used to randomly select patients according to the preassigned sample size, and their primary family caregivers were enrolled simultaneously.

The inclusion criteria were as follows: (1) older adults diagnosed with dementia by a neurologist according to ICD-11 or DSM-5 criteria, with a Clinical Dementia Rating score of 2 (moderate) or 3 (severe), and their primary family caregivers; (2) caregivers who were family members of the patient (spouse, children and their spouses, grandchildren and their spouses, or other relatives) or other relatives who had been providing continuous primary home-based care for ≥3 months, to ensure that the caregiver had established a relatively stable caregiving role and accumulated sufficient caregiving experience [44]; (3) caregivers able to provide reliable information regarding the person with dementia; and (4) willingness to participate in the study. Caregivers with severe physical or mental illnesses (e.g., cancer, heart failure, severe liver or kidney disease, schizophrenia) were excluded.

A total of 662 patient–caregiver dyads were initially sampled from the 22 hospitals. Of these, 85 dyads declined to participate, 16 had invalid questionnaires, and 32 caregivers did not meet the inclusion criteria, resulting in the exclusion of 133 dyads (non-inclusion rate: 20.1%). Ultimately, 529 patient–caregiver dyads were included in the analysis (i.e., 529 older adults with moderate-to-severe dementia and their 529 family caregivers).

### Design and data collection

This was a descriptive cross-sectional study. The patient questionnaire included items assessing sociodemographic characteristics, QoL, ADL, and depression severity, whereas the family caregiver questionnaire included items assessing sociodemographic characteristics, QoL, caregiver burden, and depression severity. Before the formal survey, all investigators received standardized training to ensure consistency and reliability in data collection. During the survey, the purpose, procedures, and significance of the study were clearly explained to participants, and informed consent was obtained from all participants before data collection. For individuals who were unable to complete the questionnaire independently, trained investigators assisted in obtaining consent to ensure accuracy and completeness. After the survey, all questionnaires were carefully reviewed, and those with more than 10% missing responses, patterned or inconsistent answers, or a completion time of less than five minutes were deemed invalid and excluded. All data were independently double-entered and cross-checked by two researchers. Logical checks, as well as reliability and validity testing, were conducted to ensure the quality of the dataset and the measurement instruments.

### Patient quality of life

The Quality of Life–Alzheimer’s Disease scale (QoL-AD) was used to assess patients’ QoL [45]. It consists of 13 items, each addressing a specific domain of QoL (e.g., physical health, energy, mood, living situation, memory, family, marriage, friends, self-assurance, household chores, fun, finances, and life as a whole), and can be grouped into four dimensions: physical health and behavioral adaptation, psychological status, living environment and social relationships, and overall life satisfaction [46]. The scale has demonstrated good psychometric properties and is suitable for use across different stages of dementia. Each item is rated on a 4-point scale from 1 (poor) to 4 (excellent), yielding a total score ranging from 13 to 52, with higher scores indicating better perceived QoL. The QoL-AD has been widely used in China, and its reliability and validity have been confirmed [47]. In this study, the Cronbach’s α coefficient for this scale was 0.876.

### Caregiver burden

Caregiver burden was assessed using the Zarit Burden Interview (ZBI) [48]. The ZBI consists of 22 items that evaluate the impact of the care recipient’s condition on the caregiver’s life and comprises two domains: (a) personal strain and (b) role strain [49]. Caregivers rate each item on a five-point scale from 0 to 4 (0 = never, 1 = rarely, 2 = sometimes, 3 = quite frequently, and 4 = nearly always). Total scores range from 0 to 88, with higher scores indicating greater caregiver burden. The caregiver burden levels are categorized as follows: Scores of ≥60 indicate severe burden, scores ranging from 40 to 59 indicate moderate burden, scores between 20 and 39 indicate mild burden, and scores of <20 indicate no burden [50]. This scale has been widely used in China, and its reliability and validity have been confirmed [51]. In this study, the Cronbach’s α coefficient for this scale was 0.904.

### Patient and caregiver depression severity

The Patient Health Questionnaire-9 (PHQ-9) was used as a self-administered screening tool to assess the presence and severity of depressive symptoms [52]. It comprises nine items grouped into two domains: emotional and somatic symptoms. The PHQ-9 aligns with the diagnostic criteria for major depression as outlined in the DSM-IV and DSM-5. Each item is scored from 0 to 3, yielding a total score ranging from 0 to 27. The total score categorizes depression severity into five levels: minimal depression (0–4), mild depression (5–9), moderate depression (10–14), moderately severe depression (15–19), and severe depression (20–27) [53]. This scale has been widely used in China, and its reliability and validity have been confirmed [54,55]. In this study, caregivers self-administered the PHQ-9, with a Cronbach’s α of 0.892; patients completed it independently when possible, or via trained interviewer-administered interviews when necessary, with a Cronbach’s α of 0.886.

### Caregiver quality of life

Caregiver QoL was assessed using the World Health Organization Quality of Life–Brief Version (WHOQoL-BREF) [56]. The scale includes 26 items categorized into four domains: environment (8 items), physical health (7 items), psychological health (6 items), and social relationships (3 items) [57]. A five-point Likert scale is used, with higher scores indicating better QoL. This scale has been widely used in China, and its reliability and validity have been confirmed [58,59]. In this study, the Cronbach’s α coefficient for this scale was 0.879.

### Patient activity of daily living

The Activity of Daily Living Scale (ADLs) was assessed using a modified 20-item scale adapted from Lawton and Brody’s 1969 instrument [26]. The scale consists of two subscales: Basic ADL and Instrumental ADL. Each item is rated on a four-point scale: 1 = “can do it independently,” 2 = “have some difficulty but can still do it independently,” 3 = “need assistance,” and 4 = “cannot do it at all.” The total ADL score ranges from 20 to 80, with higher scores indicating greater functional impairment. In this study, the scale demonstrated strong internal consistency, with a Cronbach’s α coefficient of 0.944.

### Statistical analysis

Descriptive statistics were used to describe the participants’ sociodemographic characteristics and the main study variables (patient ADL, patient QoL, patient depression severity, caregiver QoL, caregiver depression severity, and caregiver burden). Data were analyzed using IBM SPSS Statistics, version 25.0 (IBM Corp., Armonk, NY, USA) and IBM SPSS Amos, version 24.0 (IBM Corp., Armonk, NY, USA). Data quality was evaluated using Cronbach’s alpha (> 0.60), the Kaiser–Meyer–Olkin (KMO) measure of sampling adequacy (> 0.60), and Bartlett’s test of sphericity. One-way analysis of variance and independent-samples t-tests were used to examine the associations between sociodemographic characteristics and caregiver burden. The associations among patient ADL, QoL, severity, caregiver QoL, depression severity, and caregiver burden were examined using Pearson correlation coefficients. Structural equation modeling (SEM) was used to identify factors associated with caregiver burden. Variables that were significantly associated with caregiver burden in the correlation analyses were entered into the SEM, with caregiver burden specified as the dependent variable. In addition, a mediation model was specified to examine the mediating role of caregiver QoL in the relationship between the independent variables and caregiver burden. Because patient ADL, QoL, depression severity, caregiver QoL, depression severity, and caregiver burden are latent constructs that cannot be directly measured, they were modeled as latent variables in the SEM. The dimension scores of the corresponding validated instruments were included as observed indicators for these latent variables. The model was estimated using maximum likelihood. All tests were two-sided with a significance level of α = 0.05. Model fit was evaluated using multiple indices, including the chi-square/degrees of freedom ratio (CMIN/DF), goodness-of-fit index (GFI), adjusted goodness-of-fit index (AGFI), root mean square error of approximation (RMSEA), comparative fit index (CFI), and normed fit index (NFI).

### Ethical approval

This study was reviewed and approved by the Ethics Committee of Scientific Research, the First Hospital of Shanxi Medical University (Approval No. KYLL-2025–108; approved on 21 March 2025). Before starting the survey, the study objectives and procedures were explained to all participants and their designated surrogate decision-makers. Participants were adequately informed that all information would be kept strictly confidential and that no identifiable data would be disclosed. They were also informed that participation was voluntary and that they could withdraw from the study at any time. We have provided a detailed description of the above content on the first page of the questionnaire. If participants agree to take part in the study, they can proceed to the next page on their own to fill out the questionnaire.

## Results

### Descriptive statistics

A total of 529 dyads of older adults with moderate-to-severe dementia and their family caregivers were included in the study. Demographic characteristics are shown in Table 1. Among patients, the sex distribution was approximately balanced, most were between 60 and 79 years of age, 43.48% had an education level of primary school or below, 77.32% were married, 73.91% had lived with dementia for ≤2 years, and 47.64% had a monthly income of <1,000 CNY. Among caregivers, 63.52% were female, 92.25% were married, 75.43% lived with the patient, and 80.52% reported having at least one co-caregiver; more than half of the caregivers were the patient’s children or the children’s spouses. There were significant differences in caregiver burden according to the duration of dementia, patients’ monthly income, caregivers’ gender, whether they lived with the older adult, monthly additional expenses due to caregiving, and the number of assisted caregivers (Table 2).

**Table 1:** Demographic and clinical characteristics (N = 529).

Variable (patients)	Number (%)	Variable (caregivers)	Number (%)
Gender		Gender	
male	262(49.53)	male	193(36.48)
Female	267(50.47)	Female	336(63.52)
Age range (years)		Age range (years)	
60-69	216(40.83)	≤29	17(3.21)
70-79	199(37.62)	30 ~ 39	89(16.82)
80-89	101(19.09)	40 ~ 49	114(21.55)
90	13(2.46)	50 ~ 59	129(24.39)
Education		≥60	180(34.03)
Primary school or below	230(43.48)	Education	
Middle school	192(36.29)	Primary school or below	121(22.87)
Senior school	87(16.45)	Middle school	202(38.19)
College or above	20(3.78)	Senior school	126(23.82)
Marital status		College or above	80(15.12)
Married	409(77.32)	Marital status	
Divorce	13(2.46)	Married	488(92.25)
Widowed	105(19.85)	Divorce	11(2.08)
Unmarried	2(0.38)	Widowed	15(2.84)
Years of dementia		Unmarried	15(2.84)
≤2	391(73.91)	Monthly economic income(yuan)	
3 ~ 5	98(18.53)	<1000	154(29.11)
>5	40(7.56)	1000 ~ 3000	211(39.89)
Monthly economic income		3001 ~ 5000	127(24.01)
<1000	252(47.64)	>5000	37(6.99)
1000 ~ 3000	174(32.89)	Living with the patient	
3001 ~ 5000	83(15.69)	Yes	399(75.43)
>5000	20(3.78)	No	130(24.57)
		Number of assisted caregivers	
		0	103(19.47)
		1	257(48.58)
		2	133(25.14)
		≥3	36(6.81)
		Relationship between caregiver and patients	
		Spouse	171(32.33)
		Children and their spouses	294(55.58)
		Grandchildren and their spouses	9(1.7)
		Other family members	55(10.4)
		Monthly additional expenses due to caregiving	87(16.45)
		<1000	241(45.56)
		1000 ~ 3000	231(43.67)
		3001 ~ 5000	40(7.56)
		>5000	17(3.21)

**Table 2:** Differences in caregiver burden by sociodemographic characteristics (N = 529).

Characteristics	Caregiver burden	Statistical value	*P*
Years of dementia		6.356	0.002**
≤2	40.26 ± 13.73		
3 ~ 5	44.11 ± 13.12		
>5	46.70 ± 13.39		
Monthly economic income of patient			
<1000	43.46 ± 13.25	13.023	0.000**
1000 ~ 3000	41.90 ± 13.26		
3001 ~ 5000	38.30 ± 14.12		
>5000	25.60 ± 9.88		
Gender of caregiver			
male	39.84 ± 13.47	−2.065	0.039*
Female	42.39 ± 13.81		
Living with the patient			
Yes	42.48 ± 13.59	9.003	0.003**
No	38.35 ± 13.74		
Number of assisted caregivers			
0	43.42 ± 13.48	6.719	0.000**
1	42.08 ± 13.50		
2	41.29 ± 13.53		
≥3	32.06 ± 13.70		
Monthly additional expenses due to caregiving			
<1000	40.40 ± 13.34	7.143	0.000**
1000 ~ 3000	41.06 ± 13.63		
3001 ~ 5000	44.27 ± 14.29		
>5000	55.35 ± 11.92		

Only significant results have been listed.

*p-value < .05, **p-value < .01.

### Patient QoL, ADL, depression severity, caregiver depression severity, QoL, and burden

Analysis results for patient QoL, ADL, depression severity, caregiver depression severity, QoL, and burden are presented in Table 3. The QoL-AD score was 29.936 ± 7.907; the ADLs score was 48.550 ± 13.491; the PHQ-9 score for patients was 13.677 ± 6.139; the PHQ-9 score for caregivers was 13.293 ± 6.246; the WHOQoL-BREF score was 75.257 ± 14.150; and the ZBI score was 41.461 ± 13.733.

**Table 3:** Analysis results: patient QoL, ADL, depression severity, caregiver depression severity, QoL, and burden.

	Parameters	M	SD	Min	Max
patient	Quality of Life	29.936	7.907	14	49
	Activities of Daily Living	48.550	13.491	20	75
	Depression Severity	13.677	6.139	2	25
caregiver	Depression Severity	13.293	6.246	1	25
	Quality of Life	75.257	14.150	46	109
	Caregiver Burden	41.461	13.733	12	70

*M*, mean; *SD*, standard deviation.

### Correlations between Patient QoL, ADL, depression severity, caregiver depression severity, QoL, and burden

Pearson’s correlation analysis (Table 4) showed that caregiver burden was negatively correlated with patient QoL, ADL, and caregiver QoL, and positively correlated with both patient and caregiver depression severity. In addition, caregiver QoL was positively correlated with patient QoL and patient ADL, and negatively correlated with patient and caregiver depression severity.

**Table 4:** Correlations between QoL-AD, PHQ-9(patient), ADLs, WHOQoL-BREF, PHQ-9(caregiver), and ZBI.

		patient			caregiver		
		Quality of Life	Activities of Daily Living	Depression Severity	Depression Severity	Quality of Life	Burden
patient	Quality of Life	1					
	Activities of Daily Living	0.125**	1				
	Depression Severity	-0.116**	-0.313**	1			
caregiver	Depression Severity	-0.130**	-0.287**	0.375**	1		
	Quality of Life	0.145**	0.309**	-0.297**	-0.348**	1	
	Burden	-0.205**	-0.363**	0.421**	0.432**	-0.427**	1

**p < 0.01.

The purpose of this study was to explore the relationships among QoL, ADL, and depression severity in older adults with moderate-to-severe dementia, as well as family caregivers’ QoL, depression severity, and caregiver burden, and to identify the factors related to caregiver burden. In our sample, caregiver burden was generally in the mild-to-moderate range. Consistent with our hypotheses, patient QoL, ADL, and caregiver QoL exerted direct negative effects on caregiver burden, whereas the depression severity in both patients and caregivers showed direct positive effects on caregiver burden. Moreover, caregiver QoL partially mediated the associations of patient QoL, depression severity, ADL, and caregiver depression severity with caregiver burden.

### Model test

**Fig 2:**
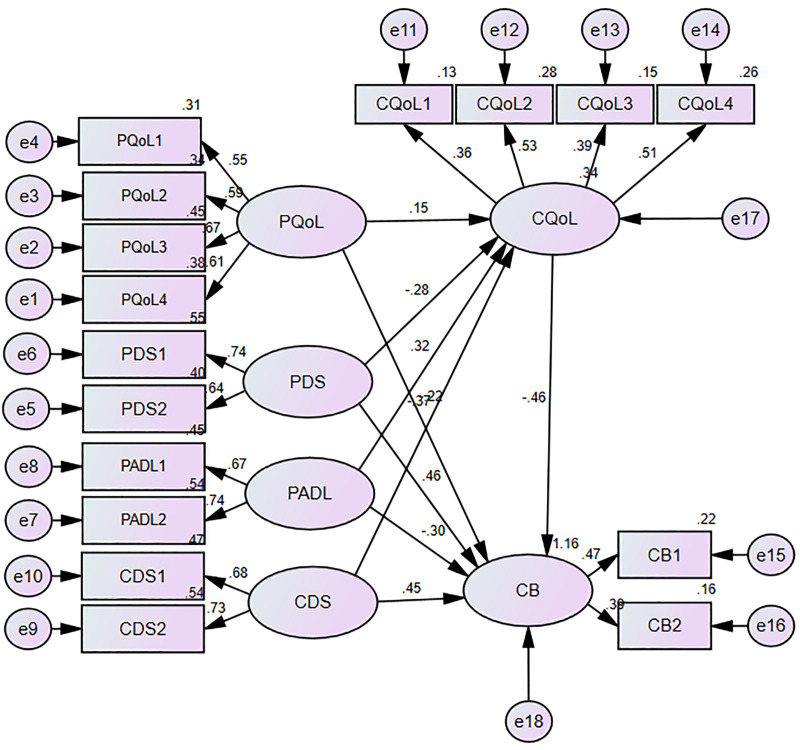
Structural equation model of influencing factors of caregiver burden. PQoL-patient quality of life; PADL-patient activities of daily living; PDS-patient depression severity; CQoL-caregiver quality of Life; CDS-caregiver depression severity; CB-caregiver burden. The oval boxes represent latent variables, while the rectangular boxes represent observed variables.

Structural equation modeling was used to test the correlations among the variables (Fig 2). The results showed that the models fit the data well (CMIN/DF = 2.844, GFI = 0.935, AGFI = 0.907, RMSEA = 0.059, CFI = 0.878, and NFI = 0.826). The standardized estimation of each path in the model is shown in Table 5. The coefficients indicated that patient QoL, ADL, and caregiver QoL had direct negative effects on caregiver burden (β = −0.222, *P* = 0.009; β = −0.297, *P* = 0.005; β = −0.464, *P* = 0.003), whereas patient and caregiver depression severity had direct positive effects on caregiver burden (β = 0.456, *P* < 0.001; β = 0.453, *P* < 0.001). Meanwhile, caregiver QoL partially mediated the relationships among patient QoL (β=−0.071; 95% confidence interval, −0.193, −0.008; *P* = 0.023), depression severity (β = 0.131; 95% confidence interval, 0.039, 0.312; *P* = 0.004), ADL (β = −0.15; 95% confidence interval, −0.345, −0.044; *P* = 0.006), and caregiver depression severity (β = 0.17; 95% confidence interval, 0.052, 0.412; *P* = 0.006) and caregiver burden.

**Table 5:** Standardized estimation of each path in the model.

Path			Path coefficient	Estimates	S.E.	C.R.	*P*
Patient Quality of Life	→	Caregiver 1uality of life	0.152	0.304	0.144	2.115	0.034
Patient depression severity	→	Caregiver quality of life	−0.282	−0.248	0.074	−3.327	***
Patient activities of daily Living	→	Caregiver quality of life	0.324	0.106	0.03	3.518	***
Caregiver depression severity	→	Caregiver quality of life	−0.366	−0.279	0.071	−3.926	***
Patient quality of Life	→	Caregiver burden	−0.222	−0.865	0.333	−2.597	0.009
Patient depression severity	→	Caregiver burden	0.456	0.786	0.181	4.347	***
Patient activities of daily living	→	Caregiver burden	−0.297	−0.191	0.068	−2.824	0.005
Caregiver quality of life	→	Caregiver burden	−0.464	−0.909	0.308	−2.953	0.003
Caregiver Depression Severity	→	Caregiver Burden	0.453	0.677	0.168	4.023	***

***p < 0.001; S.E., standard error; C.R., critical ratios.

## Discussion

The purpose of this study was to explore the relationships among QoL, ADL, and depression severity in older adults with moderate-to-severe dementia, as well as family caregivers’ QoL, depression severity, and caregiver burden, and to identify the factors related to caregiver burden. In our sample, caregiver burden was generally in the mild-to-moderate range. Consistent with our hypotheses, patient QoL, ADL, and caregiver QoL exerted direct negative effects on caregiver burden, whereas the depression severity in both patients and caregivers showed direct positive effects on caregiver burden. Moreover, caregiver QoL partially mediated the associations of patient QoL, depression severity, ADL, and caregiver depression severity with caregiver burden.

In our study, caregiver burden was higher than that reported in several Asian countries [60] (e.g., Thailand, the Philippines, Singapore, and South Korea) as well as in Western countries [61], which may be attributable to substantial differences in the social environment, cultural traditions, and health-care policies. In China, influenced by the Confucian value of filial piety, caregivers often perceive caring for parents with dementia as a responsibility and moral obligation [62]; Consequently, they may be reluctant to seek external assistance and may delay institutionalization of the patient [63]. Meanwhile, traditional Chinese culture emphasizes endurance and self-sacrifice, which may lead family caregivers to overlook their own psychological strains and needs [64]. As a result, available psychological resources may be deprioritized and underutilized, ultimately exacerbating caregiver burden. In addition, China is still developing and refining community-based eldercare and professional nursing services, limiting access to social support, caregiving resources, and policy protections for care providers, which may further intensify caregiver burden [65,66]. However, compared with findings from domestic studies, the burden scores in our sample were lower than those reported in Shanghai [67] and Hangzhou [44], potentially due to differences in dementia severity among included participants and regional disparities in economic conditions.

The characteristics of patients, caregivers, and families are considered to be crucial to caregiver burden. For example, Kang et al. [68] reported that a longer dementia disease course is associated with greater subjective burden among caregivers, supporting the “wear-and-tear” hypothesis whereby prolonged caregiving increases the likelihood of adverse outcomes [69]. In the present study, economic resources were significantly associated with caregiver burden: lower patient monthly income and higher additional monthly family expenditures attributable to caregiving were both linked to greater burden. Alhasan et al. [70] further demonstrated that persons with dementia living in low-income communities have a significantly higher prevalence of neuropsychiatric symptoms (e.g., depression, anxiety, hallucinations). These behavioral and psychological symptoms constitute a major source of caregiver stress and can substantially increase the complexity of daily care and emotional exhaustion. Concurrently, the financial strain imposed by dementia care represents a core dimension of caregiver burden, with economic difficulties frequently identified as a central component of the burden construct [71]. With respect to caregiver sex, female caregivers tend to experience higher levels of burden when caring for older adults with dementia. A systematic review and meta-analysis encompassing 47 studies across 24 countries showed that female family caregivers report significantly greater burden than males; this pattern is particularly pronounced in Asian countries and high-income settings [72]. Moreover, women are more likely to employ emotion-focused coping strategies, which have been associated with higher distress [73]. Female caregivers may also exhibit more negative attitudes and emotional responses—such as social restrictiveness, anxiety, and aggressiveness—factors that are themselves related to elevated burden [74]. Our findings also indicated that caregivers co-residing with the care recipient experienced higher burden, potentially because co-residence entails substantially greater time investment and blurs temporal and spatial boundaries, thereby reducing personal space and increasing psychological load [75]. In addition, social support has been consistently shown to be a key protective factor against caregiver burden. In our sample, 80.53% of caregivers reported having auxiliary caregiving personnel, and caregiver burden decreased as the number of auxiliary caregivers increased, consistent with findings by Clyburn et al. [76] Hashima et al. [77] further reported that caregivers who perceive stronger social support (from family, friends, or significant others) are less likely to report burden as well as depressive and anxiety symptoms, while demonstrating better QoL and higher caregiving self-efficacy.

The present study demonstrated a negative association between patients’ ADL and caregiver burden; specifically, poorer ADL functioning was related to greater caregiver burden. Razani et al. [78] reported significant and relatively strong inverse correlations between patients’ ADL performance and caregivers’ time-dependence burden, developmental burden, and psychological strains (e.g., hostility). These findings suggest that as patients’ capacity to independently perform everyday activities (such as financial management, transportation, and personal hygiene) declines, caregivers must devote substantially more time and effort to assisting. This increased caregiving demand can constrain caregivers’ personal lives and opportunities for development, thereby contributing to a heavier perceived burden and heightened psychological strain. Further evidence indicates that the impact of ADL impairment on caregiver burden varies by the type of functional limitation. Compared with basic ADL, impairments in instrumental ADL (e.g., managing finances, shopping, and medication management) may be more closely linked to caregiver burden. In particular, Razani et al. found that deficits in financial management were the strongest predictor of caregivers’ time-dependent burden and hostile affect. This may be because instrumental ADL tasks rely on more complex cognitive processes, including executive function; deterioration in these abilities not only signals disease progression but also imposes greater demands on caregivers for supervision, monitoring, and decision-making [79]. Notably, even at the stage of mild cognitive impairment (MCI), patients may already exhibit complex ADL deficits, and caregivers of individuals with MCI have been reported to experience significantly higher burden than caregivers of patients with other chronic diseases [80]. This suggests that ADL-related burden may emerge early in the dementia trajectory [81]. Accordingly, early attention to and systematic assessment of ADL functioning in persons with dementia are essential. Regular functional assessment can help identify patients with rapid decline and enable the timely provision of targeted support and interventions for caregivers [82]. Rehabilitation programs aimed at maintaining or improving patients’ ADL abilities, together with training caregivers in effective strategies to manage ADL-related impairments, may represent important approaches to reducing caregiver burden and improving caregivers’ mental health [78,82].

Improvements in patients’ QoL were directly associated with reduced caregiver burden, consistent with prior evidence. This inverse relationship may operate through three main pathways. First, better patient QoL is often accompanied by a reduction in neuropsychiatric symptoms (e.g., depression, anxiety, and apathy), which can directly lessen caregivers’ exposure to challenging behaviors and the complexity of symptom management [83,84]. Evidence suggests that when patients maintain a more favorable emotional state and functional level, caregivers report significantly lower perceived burdens [83]. Second, enhanced patient QoL frequently reflects preserved capacity in ADL, thereby reducing the amount and intensity of hands-on assistance required from caregivers [85]. Most importantly, patients with higher QoL tend to retain better social functioning and interpersonal engagement, allowing the caregiving context to preserve more positive relational elements rather than devolving into a purely unidirectional “caregiver–care recipient” dynamic [86,87]. Notably, this association is influenced by several moderating factors. Studies suggest that the burden-buffering effect of patient QoL is more pronounced when caregivers have greater disease-related knowledge and higher educational attainment [88]. In addition, disease stage appears to moderate this relationship, with the negative association between QoL and caregiver burden being stronger among individuals with mild-to-moderate dementia [83,89]. From a clinical perspective, these findings underscore the importance of adopting a holistic intervention approach that conceptualizes patient QoL and caregiver burden as components of an interconnected system rather than isolated outcomes. Recommended strategies include optimizing the management of patients’ neuropsychiatric symptoms, implementing activity planning to maintain functional abilities, and providing educational support to strengthen caregivers’ disease knowledge and coping skills [88,90].

The present study found a significant positive association between depression severity in persons with dementia and caregiver burden. Specifically, patients’ depressive symptoms emerged as an important predictor of increased caregiver burden. This relationship can be interpreted within the stress–appraisal framework: patients’ behavioral problems, including neuropsychiatric symptoms such as depression, are perceived by caregivers as major stressors, which subsequently shape caregivers’ subjective appraisal of burden [76]. Notably, depression-related caregiving burden appears to be prevalent across caregivers of different dementia subtypes (e.g., Alzheimer’s disease, dementia with Lewy bodies) [91,92]. However, the magnitude of the association may vary by dementia etiology and the specific symptom profile (e.g., the presence of delusions) [91]. These findings underscore that clinical management and support services should address not only cognitive decline in dementia but also systematically assess and treat affective symptoms—particularly depression—as a key component of efforts to reduce caregiver burden and safeguard caregivers’ psychological and physical well-being [76,82].

Concurrently, the present study identified a significant positive association between caregivers’ depressive symptoms and caregiver burden. Prolonged exposure to challenges such as patients’ cognitive decline, fluctuations in behavioral and psychological symptoms, increasing functional dependence, and the intensive demands of daily care can foster persistent tension and helplessness in caregivers. Depressive affect may further reduce stress tolerance and perceived sense of control, leading caregivers to appraise caregiving tasks as more “onerous” and “difficult to manage,” thereby manifesting as higher perceived burden. Notably, caregiver depression and burden frequently co-occur, and sex differences have been reported. Evidence suggests that female caregivers not only exhibit more pronounced depressive symptoms but also experience significantly greater caregiving burden than male caregivers [93]. Mechanistically, depressive symptoms may exacerbate caregiver burden through multiple pathways: first, depression can undermine psychological resilience and problem-solving capacity [94]; second, it is often accompanied by sleep disturbances and somatic symptoms [95], which further deplete caregivers’ physiological coping resources; and third, depression may impair caregivers’ ability to seek and mobilize social support, creating a self-reinforcing vicious cycle. These findings indicate that clinical interventions should place particular emphasis on the identification and management of depressive symptoms in caregivers. Early detection and timely treatment may help interrupt the escalation of burden and prevent the development of maladaptive cycles [96]. Future research should further examine personalized interventions targeting caregiver depression to alleviate caregiver burden and improve overall care quality.

Caregivers’ QoL not only exerts a direct effect on caregiver burden but may also partially mediate the associations described above. First, caregiver QoL has direct predictive value for perceived burden. Although most studies have focused on the impact of burden on QoL [97,98], the relationship is inherently bidirectional and dynamic. Lower caregiver QoL may stem from poor health, financial strain, or insufficient social support—factors that can independently intensify emotional, physical, and economic stress and thereby increase caregiver burden [97]. Conversely, higher caregiver QoL may enhance coping capacity and reduce the subjective appraisal of burden [99,100]. For example, among caregivers of stroke survivors, poorer self-rated health and lower household income have been associated with reduced QoL, which in turn contributes to greater perceived burden [97]. Similarly, in epilepsy caregiving, burden is negatively correlated with caregiver QoL, suggesting that improving QoL may directly alleviate burden [101].

Second, the present study found that caregiver QoL partially mediated the relationships between caregiver burden and patients’ ADL, QoL, depression severity, and caregivers’ depression severity. Caregiver QoL is widely conceptualized as a comprehensive outcome reflecting overall well-being [102], encompassing physical health, psychological status, social functioning, and role adaptation. When patients experience functional decline, greater depressive symptoms, or reduced QoL, caregivers typically must invest more time and effort, while facing heightened emotional exhaustion and coping demands; these stressors can diminish caregiver QoL and subsequently exacerbate subjective caregiver burden. Moreover, caregivers with higher levels of depressive symptoms may experience reduced energy, more negative cognitions, and constrained coping strategies [103], which lower their tolerance for caregiving demands and increase the likelihood of appraising care tasks as onerous and uncontrollable. This process can directly impair caregiver QoL and further amplify perceived burden. Therefore, intervention planning should not only prioritize patient-focused components (e.g., functional rehabilitation, symptom management, and QoL enhancement) but also explicitly target improvements in caregiver QoL. Potential approaches include caregiving skills training, stress management and psychological support, peer support, and respite services to improve sleep, alleviate depressive symptoms, strengthen coping self-efficacy, and enhance social support—thereby reducing caregiver burden at its source and promoting a more adaptive and sustainable caregiving dynamic.

Finally, prior studies have shown that caregiver burden increases substantially when persons with dementia also have additional chronic conditions. Comorbidity is highly prevalent in dementia and directly increases treatment complexity and the demands of medication management. For example, people with dementia have been reported to live, on average, with approximately 5–6 chronic diseases and to use 4–5 medications, requiring caregivers to devote considerable time to coordinating medical care [104]. Comorbidity is also associated with a higher risk of potentially avoidable hospitalizations, particularly in the context of suboptimal control of conditions such as diabetes and hypertension, thereby intensifying caregivers’ physical workload and financial strain [105]. Moreover, the overall burden of multimorbidity is closely linked to poorer caregiver QoL and greater out-of-pocket expenditures [106]. Collectively, these findings suggest that comorbidities in dementia exacerbate family caregiver burden through heightened medical complexity, increased risk of acute events, and amplified psychosocial and economic demands. Although this factor was not examined in the present study, the burden imposed by comorbidities among persons with dementia on family caregivers should not be overlooked.

### Limitations

This study has several limitations. First, its cross-sectional design precludes causal inference. Second, because participants were recruited from hospital-based, care-seeking patients, selection bias may be present. Third, the PHQ-9 may have limited validity in patients with severe dementia. Finally, restricting inclusion to caregivers with ≥3 months of caregiving may under-represent those with shorter caregiving exposure. Future studies should incorporate physiological assessments to examine these factors from a biological perspective, as they are often associated with adverse health outcomes.

## Conclusions

This study used a structural equation modeling framework to examine the mechanisms through which patient- and caregiver-related factors influence caregiver burden in family caregivers of older adults with moderate-to-severe dementia. The findings suggest that poorer patient QoL, greater functional impairment, and higher levels of depressive symptoms in both patients and caregivers are associated with greater caregiver burden. In contrast, caregiver QoL shows a significant protective effect and partially mediates these associations. Therefore, reducing caregiver burden requires integrated, dyadic care that addresses depressive symptoms and functional decline in persons with dementia, prioritizes caregiver QoL, and targets support to high-risk families, alongside policy efforts to expand community-based services (notably psychological counseling and caregiver training) and strengthen protections that lower barriers to service use.

## Supporting information

S1 File Data. (XLSX)

## Abbreviations:

QoL: quality of life
ADL: Activities of Daily Living
ZBI: Zarit Caregiver Burden Interview
QoL-AD: Quality of Life-Alzheimer′s Disease
PHQ-9: Patient Health Questionnaire-9
ADLs: Activity of Daily Living Scale
WHOQoL-BREF: World Health Organization Quality of Life
SEM: Structural equation modeling
